# Gold Nanoparticles Functionalized with 2-Thiouracil for Antiproliferative and Photothermal Therapies in Breast Cancer Cells

**DOI:** 10.3390/molecules28114453

**Published:** 2023-05-31

**Authors:** Génesis Lorenzana-Vázquez, Ioana Pavel, Enrique Meléndez

**Affiliations:** 1 Department of Chemistry, University of Puerto Rico, Mayaguez Campus, Mayaguez, PR 00681, USA; genesis.lorenzana@upr.edu; 2 Department of Physical and Environmental Sciences, Texas A&M University—Corpus Christi, Corpus Christi, TX 78412, USA; ioana.pavel@tamucc.edu

**Keywords:** gold nanoparticles, AuNPs, MDA-MB-231, 2-TU-AuNPs, Transmission Electron Microscopy (TEM), 3-[4,5-dimethylthiazol-2-yl]-2,5 diphenyl tetrazolium bromide (MTT)

## Abstract

Nanoparticles have been used to transport drugs to various body parts to treat cancer. Our interest is in gold nanoparticles (AuNPs) since they have the capacity to absorb light and convert it to heat, inducing cellular damage. This property is known as photothermal therapy (PTT) and has been studied in cancer treatment. In the present study, biocompatible citrate-reduced AuNPs were functionalized with a biologically active compound, 2-thiouracil (2-TU), of potential anticancer activity. Both the unfunctionalized (AuNPs) and functionalized (2-TU-AuNPs) were purified and characterized by UV–Vis absorption spectrophotometry, Zeta potential, and Transmission Electron Microscopy. Results showed monodispersed, spherical AuNPs with a mean core diameter of 20 ± 2 nm, a surface charge of −38 ± 5 mV, and a localized surface plasmon resonance peak at 520 nm. As a result of functionalization, the mean core diameter of 2-TU-AuNPs increased to 24 ± 4 nm, and the surface charge increased to −14 ± 1 mV. The functionalization of AuNPs and the load efficiency were further established through Raman spectroscopy and UV–Vis absorption spectrophotometry. The antiproliferative activities of AuNPs, 2-TU and 2-TU-AuNPs were examined by a 3-[4,5-dimethylthiazol-2-yl]-2,5 diphenyl tetrazolium bromide (MTT) assay in the MDA-MB-231 breast cancer cell line. It was established that AuNPs significantly enhanced the antiproliferative activity of 2-TU. Furthermore, the irradiation of the samples with visible light at 520 nm decreased the half-maximal inhibitory concentration by a factor of 2. Thus, the 2-TU drug concentration and its side effect during treatments could be significantly reduced by synergistically exploiting the antiproliferative activity of 2-TU loaded onto AuNPs and the PTT effect of AuNPs.

## 1. Introduction

Breast cancer is caused by the uncontrolled growth of breast cells. According to the World Health Organization, breast cancer was the most common cancer globally in 2021. Statistics have shown that one in eight U.S. women develops breast cancer. In cancer therapy, there are two major problems: drug resistance that the body develops and damage caused by the drug to non-malignant cells. Nanoparticles (NPs) can play a significant role in the treatment of cancer because their size allows them to percolate across the cancer cell pores. Since the gaps between vascular cells in solid tumors are wider (200 nm–1.2 µm in size) compared to normal tissues (<10 nm in size), NPs that carry drugs can easily pass through the leaky vascular walls of the tumor and accumulate in the tumor regions. This occurs due to the enhanced permeability and retention (EPR) effect [1]. Nanoparticles are among the smallest drug delivery moiety that can still behave as an entity in terms of properties. Nanoparticles can improve the solubility, stability and efficacy of drugs that are loaded on them, thereby decreasing their toxicity and achieving stable therapeutic levels for long time periods.

Gold (Au) in its natural form is considered an inert noble metal with therapeutic and medicinal properties [2]. In recent years, gold nanoparticles (AuNPs) have been studied for diagnosis and cancer therapy. AuNPs are 600 times more permeable to cancer cells than to normal, healthy cells [3]. Additionally, AuNPs can transport optimal concentrations of small drug molecules onto their surface. As a result, AuNPs find numerous biomedical applications in thermal ablation, sensor systems, bioimaging techniques, vaccine development, photothermal therapy, radiotherapy enhancement treatments, and drug delivery [4,5]. Photothermal therapy (PTT) is a non-invasive way to eradicate cancer with minimum toxic effects in comparison with other cancer therapies. The photothermal effect consists of the conversion of near-infrared (NIR) light to heat. This therapy may kill cancer selectively because cancer cells are more sensitive to heat than healthy cells [6,7]. The tumor environment is more hypoxic, acidic, and nutrient-deficient than normal tissues, which increases the sensitivity of cancer cells to heat [8]. AuNPs exhibit the localized surface plasmon resonance (LSPR) property, a unique phenomenon to plasmonic nanoparticles. This property leads to strong electromagnetic fields at the particle surface and consequently enhances all the radiative properties [9]. Although visible (VIS) light has been applied in the surgical ablation of cells, visible irradiation using AuNPs found fewer applications to cancer therapy than NIR. Visible light penetration into the tissue is reduced to less than a millimeter, which provides higher precision in certain medical procedures [10]. For instance, green lasers (495–570 nm) have been used for decades as a safe tool for tissue ablation in ocular surgeries [11]. Spherical AuNPs with diameters ranging from 10 to 30 nm are ideal photothermal agents for such biomedical applications. They present a characteristic LSPR band around 520 nm in the visible region of the spectrum with an efficient light-to-heat conversion [12,13].

AuNPs are biocompatible and have a strong affinity for biomolecules containing amines, disulfide bonds, and thiol groups. Compounds containing these moieties can be easily loaded onto the surface of AuNPs. Nitrogenous-based derivatives such as 6-mercaptopurine (6-MP) and 6-thioguanine (6-TG) are U.S. Food and Drug Administration (FDA)-approved drugs for cancer treatment [14]. 6-MP is used in the clinical treatment of human acute lymphoblastic leukemia, systemic lupus erythematosus and rheumatoid arthritis [15,16]. 6-TG is used for the treatment of leukemia, breast cancer and brain tumors [17]. However, patients who undergo long-term treatment with thiopurine drugs and sun exposure have a 50–200-fold increased incidence of skin cancer. A new method to eliminate this phototoxicity was to load the thiolated drugs onto the AuNPs surface [18] to enhance their antiproliferative activity. This is related to the reduction of the drug concentration, which decreases the side effects.

Thiouracils possess significant biological and pharmacological importance, particularly as potential anticancer drugs [19]. They are effective in controlling tumor growth, inhibiting the proliferation of viruses and bacteria, treating hyperthyroidism, and have demonstrated antifungal activity [20]. Notably, 2-thiouracil (2-TU) has been identified as a significant biochemical ligand [21]. 2-TU exhibits chemotherapeutic activity in skin cancer by effectively incorporating it into nucleic acids, thereby hindering the growth of melanoma tumors [22]. Furthermore, certain 2-thio derivatives of uracil exhibit cytotoxic activity [23].

**Figure 1:**
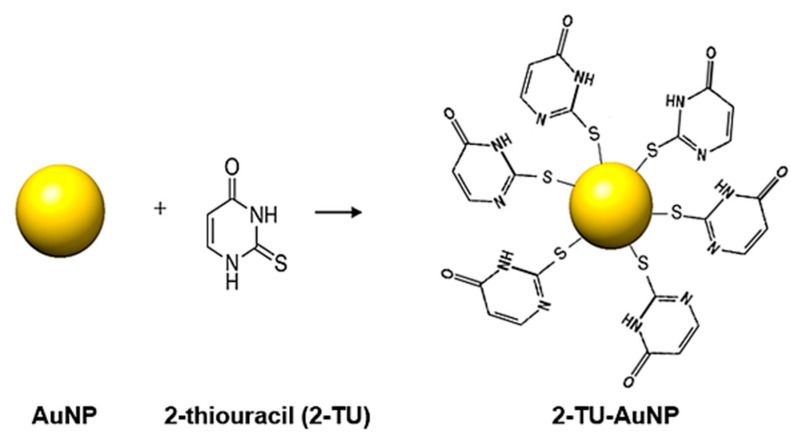
Surface functionalization of AuNPs with 2-thiouracil (2-TU).

In the present study, biocompatible citrate-reduced AuNPs were synthesized and functionalized with 2-TU via a covalent thiol-gold interaction (Figure 1). It was hypothesized that 2-TU-AuNPs would exhibit concurrent PTT in the VIS range and improved antiproliferative activities when compared to 2-TU and AuNPs alone. Thus, the PTT capabilities of both unfunctionalized (AuNPs) and functionalized nanoparticles (2-TU-AuNPs) were studied by irradiation with visible green light (520 nm) of clinical relevance, and their antiproliferative activities were established in a hormone-independent breast cancer cell line, MDA-MB-231, using an MTT assay. This cell line represents a triple-negative breast cancer with a poor prognosis, and 2-TU-AuNP is an efficient alternative drug to treat this type of aggressive cancer.

## 2. Results and Discussion

### 2.1. Characterization of Gold Nanoparticles

**Figure 2:**
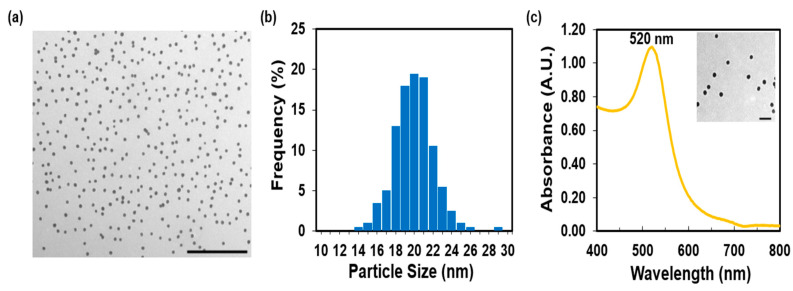
Characterization of the unfunctionalized AuNPs: (**a**) TEM image displaying in black the relatively monodispersed, spherical AuNPs (scale bar is 500 nm). (**b**) TEM size histogram showing the narrow size distribution of AuNPs in the 10–30 nm range. (**c**) UV–Vis absorption spectrum revealing an LSPR peak at 520 nm. The inset shows a TEM image with a scale bar of 100 nm.

The TEM image (Figure 2a) and histogram (Figure 2b) showed that the AuNP colloid consists of monodispersed, spherical nanoparticles of a mean core diameter of 20 ± 2 nm. The UV–Vis absorption spectrum of the citrate-stabilized AuNPs exhibited a sharp, localized surface plasmon resonance peak (LSPR) at 520 nm (Figure 2c). The concentration of the synthesized AuNPs was estimated to be 3 nM based on the absorbance of the LSPR peak and the value of the molar extinction coefficient (3.67 × 10^8^ M^−1^ cm^−1^) for this type of AuNPs [24]. The pH of AuNPs was approximately 5–6, and the ionic strength was 0.006 M. The measurement of the hydrodynamic diameter obtained from the Zetasizer Nano was 32 ± 3 nm. Zeta potential measurements confirmed the aqueous stability of the colloidal AuNPs and the presence of negative charges on the surface of AuNPs (−38 ± 5 mV). The negative charge is due to the citrate capping agents, which stem from the reducing and stabilizing agent, sodium citrate dihydrate.

**Figure 3:**
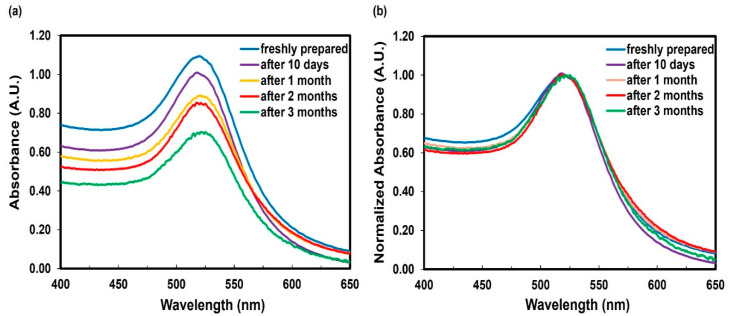
Time evolution of the LSPR peak of unfunctionalized AuNPs: (**a**) absorbance vs. wavelength (nm) and (**b**) normalized absorbance vs. wavelength (nm).

Colloidal AuNPs remained stable for over three months at room temperature. The stability was confirmed through time-dependent measurements of the LSPR band at ~520 nm of the colloidal suspension. A decrease in absorbance is observed due to the reduced concentration of AuNPs resulting from their adherence to the glass container during storage (Figure 3a). However, the absorbance was normalized to specifically examine the position and shape of the LSPR band (Figure 3b). If NPs aggregation occurs, clusters are formed, causing a noticeable shift towards longer wavelengths and often resulting in changes to the peak shape. After three months, the LSPR band exhibited a minimal shift of 5 nm units.

### 2.2. Characterization of Functionalized Gold Nanoparticles

**Figure 4:**
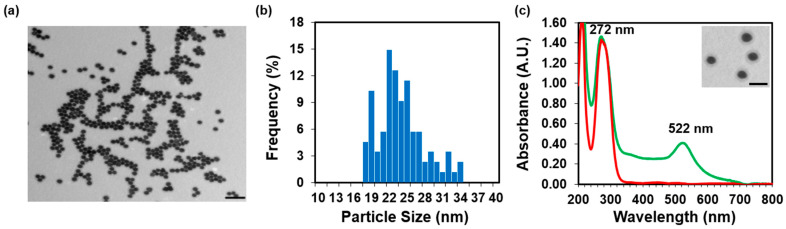
Characterization of the functionalized 2-TU-AuNPs: (**a**) TEM image displaying in black the relatively monodispersed, spherical 2-TU-AuNPs (scale bar is 100 nm). (**b**) TEM size histogram showing the narrow size distribution of AuNPs in the 15–35 nm range. (**c**) UV–Vis absorption spectra of the 2-TU in solution (red line) and 2-TU-AuNPs (green line) confirming the functionalization of AuNPs with 2-TU. The inset shows a TEM image with a scale bar of 25 nm.

The loading of AuNPs with 2-TU was confirmed through TEM, UV–Vis absorption spectrophotometry, Raman and Zetasizer measurements. In the TEM image, the AuNPs functionalized with 2-TU appeared spherical, monodispersed, and had a halo (2-TU) around them (Figure 4a). The mean core diameter of these 2-TU-AuNPs increased to 24 ± 4 nm upon the functionalization with 2-TU (Figure 4b). The main absorption peak of 2-TU corresponding to the electronic transition π–π* of the two carbonyl and thiocarbonyl groups [25] was detected around 270 nm. Both the LSPR peak characteristic to the formation of AuNPs at 522 nm and the fingerprint band of 2-TU were present in the UV–Vis absorption spectrum of 2-TU-AuNPs (Figure 4c).

UV–Vis spectroscopy was also used to estimate the concentration of 2-TU functionalized on AuNPs. Absorbance measurements were taken at various concentrations of 2-TU solutions. A calibration curve (Figure S1 of Supplementary Information) was constructed to determine the extinction coefficient of 2-TU (ε = 1.4 × 10^4^ M^−1^ cm^−1^). After purifying the 2-TU-AuNPs, the concentration of 2-TU on the surface of AuNPs was determined to be 0.15 ± 0.02 mM using the Beer–Lambert law. To further confirm the drug loading and estimate the drug loading efficiency (LE), absorbance measurements of functionalized 2-TU-AuNPs were carried out in the 200–800 nm spectral range. The AuNP colloid samples were centrifuged after the functionalization with 2-TU, and the UV–Vis absorption spectrum of the supernatant was used to determine the amount of unbound 2-TU. The loading efficiency for 2-TU-AuNPs was then estimated to be ~32% using the following Equation (1):

(1) $$\%\;\mathrm{LE}=\frac{actual\;drug\;in\;AuNPs}{drug\;added\;initially}\;\times \;100$$

**Figure 5:**
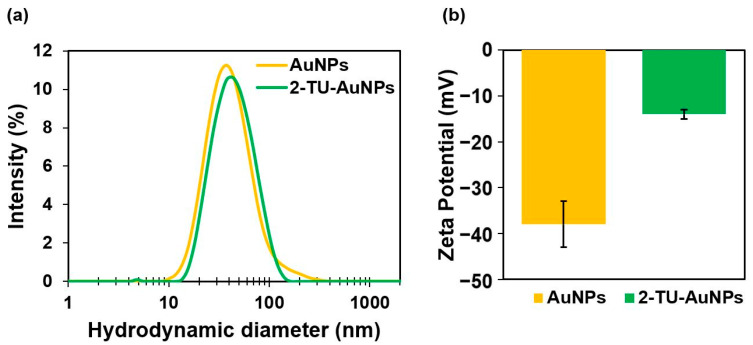
Size and Zeta potential results of AuNPs and 2-TU-AuNPs obtained from the Zetasizer Nano measurements: (**a**) hydrodynamic diameter and (**b**) zeta potential. The error bars correspond to the standard deviations.

Even though it has been argued that the citrate on the surface of AuNPs was highly resistant to displacement by thiol groups, it was demonstrated that organothiol compounds could indeed displace citrate from AuNPs [26]. After functionalization, Zetasizer results showed an increase in the hydrodynamic diameter, shifting from 32 ± 3 nm to 45 ± 5 nm (Figure 5a) due to the attachments of 2-TU molecules. The Z-potential results also indicated a surface charge increase from −38 ± 5 mV to −14 ± 1 mV (Figure 5b). This Z-potential value confirmed that the negative charge of the citrate ions was significantly reduced, demonstrating the displacement of citrate ions by the monolayer of 2-TU.

Raman measurements were conducted in the range of 300–1300 cm^−1^ for 2-TU and 2-TU-AuNPs (Figure S2 of Supplementary Information). The broad peak observed at 445 cm^−1^ and a shoulder at 355 cm^−1^ for the 2-TU-AuNPs could indicate the presence of a gold ligand bonding interaction. The metal–sulfur bond generates a peak below 500 cm^−1^ [27] which can be attributed to the gold-sulfur bond, confirming the functionalization. The Raman shifts observed between 600–800 cm^−1^ have been assigned to the carbon-sulfur bond [28] present in both molecules. The peak at approximately 1218 cm^−1^ indicates the C=S bond [29], which is only present in the 2-TU molecule.

### 2.3. Concentration of Gold Nanoparticles

**Figure 6:**
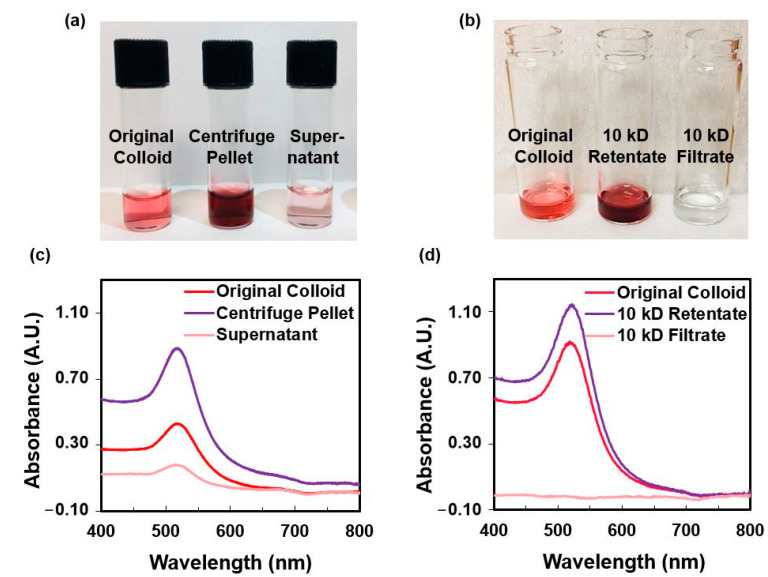
Comparison of the centrifugation and filtration capabilities in recovering and purifying the colloidal AuNPs. (**a**) Images of the vials containing the original colloid, the centrifuged pellet containing concentrated AuNPs, and the supernatant (from left to right). (**b**) Images of the vials containing the original colloid, the 10 kD retentate containing most of the recovered, concentrated AuNPs, and the 10 kD filtrate consisting mostly of water and excess reagents (from left to right). (**c**) UV–Vis absorption spectrum for the centrifugation method. (**d**) UV–Vis absorption spectrum for the filtration method.

To concentrate the colloidal AuNPs and 2-TU-AuNPs, centrifugation and tangential flow filtration were comparatively performed. After centrifugation at 13,000 rpm (15,700× *g*) for 20 min, the supernatant had a light pink color (Figure 6a), which is indicative of less efficient recovery of AuNPs. The UV–Vis absorption spectrum of the supernatant confirmed the nanomaterial presence through the LSPR peak at ~520 nm (Figure 6c). After filtration with a 10 kD filter for about 10 min, the resulting filtrate was clear (Figure 6b), and the absence of AuNPs was confirmed by UV–Vis absorption spectrophotometry (Figure 6d). Thus, filtration was selected for the concentration and purification of nanoparticles before their subsequent use in antiproliferative studies.

### 2.4. Antiproliferation Studies of Irradiated vs. Non-Irradiated Nanoparticles

**Figure 7:**
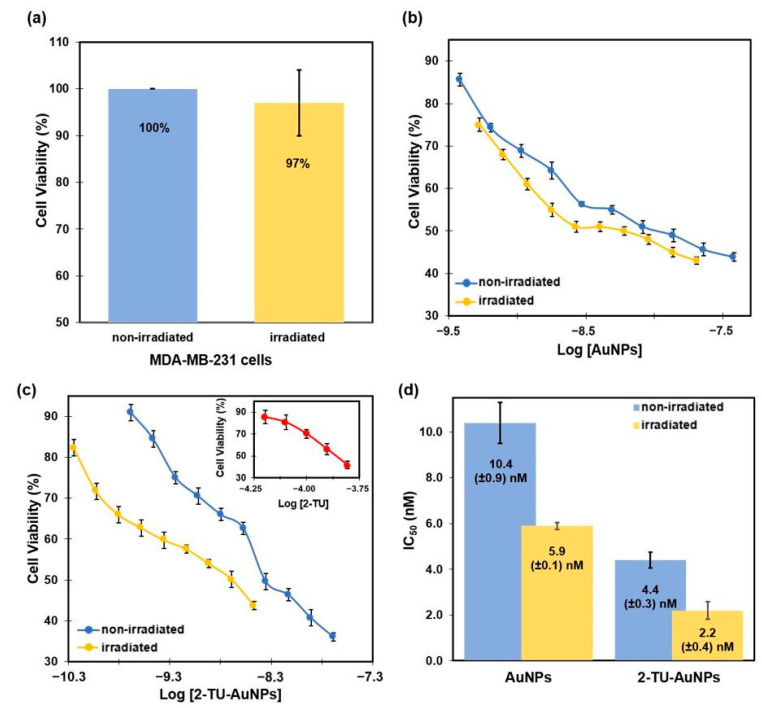
Results obtained from antiproliferation studies: (**a**) Effect of the radiation on MDA-MB-231 cells. (**b**) Comparison of the cell viabilities for non-irradiated and irradiated AuNPs at different concentrations. (**c**) Comparison of the cell viabilities for non-irradiated and irradiated 2-TU-AuNPs at different concentrations. The inset shows cell viabilities at different concentrations of 2-TU. (**d**) IC_50_ values for the non-irradiated versus the irradiated AuNPs and 2-TU-AuNPs in breast cancer cells (MDA-MB-231). The error bars correspond to the standard deviations.

The effect of radiation was initially studied in the MDA-MB-231 breast cancer cell line without AuNPs (Figure 7a). The ANOVA results (*p*-value = 0.73, >0.05) indicated no significant effect. Cells exposed to radiation did not exhibit a significant decrease in viability. This observation clearly suggests that the presence of AuNPs is necessary for the photothermal conversion of light, which ultimately induces cell death. The antiproliferative activities of AuNPs, 2-TU, and 2-TU-AuNPs (Figure 7b,c) were investigated against MDA-MB-231 cells by calculating the half-maximal inhibitory concentration (IC_50_). The IC_50_ concentration was determined for the concentration at which 50% cell viability was observed compared to that of control cells. The IC_50_ values corresponding to the non-irradiated AuNPs and the irradiated AuNPs were 10.4 ± 0.9 nM and 5.9 ± 0.1 nM, respectively, while the IC_50_ values for the non-irradiated 2-TU-AuNPs and the irradiated 2-TU-AuNPs were 4.4 ± 0.3 nM and 2.2 ± 0.4 nM, respectively (Figure 7). From the analysis, it is apparent that AuNPs showed cytotoxicity on unirradiated cells. A study found that citrate-capped AuNPs with a spherical shape and a size of 25–30 nm decreased the proliferation of MDA-MB-231 cells in the cytotoxicity assay. The researchers observed that, even in the absence of irradiation, AuNPs induced oxidative stress responsible for cell death in breast cancer cells but not in normal breast cells [30]. The IC_50_ value associated with 2-TU was 0.14 ± 0.01 mM.

In general, it can be noted that the irradiated nanoparticles have a 43–50% enhancement in antiproliferative activity when compared to the non-irradiated nanoparticles. In addition, non-irradiated AuNPs loaded with the biologically active compound, 2-TU-AuNPs, exhibited enhanced antiproliferative activity by 58% compared to unfunctionalized AuNPs and six orders of magnitude for 2-TU. Overall, the 2-TU-loaded and irradiated AuNPs have greater antiproliferative activities. Thus, combining photothermal therapy (PTT) with anticancer compounds loaded onto AuNPs lowered the amount of drug needed for therapy by 43–50%.

In a similar study, researchers functionalized the surface of AuNPs with 6-thioguanine (6-TG) and evaluated the effectiveness of both 6-TG and 6-TG-AuNPs against breast cancer cells. The results demonstrated that loading 6-TG onto the surface of AuNPs resulted in a greater cytotoxic effect compared to using 6-TG alone, as indicated by the IC_50_ values. This improved efficacy enabled the use of lower concentrations of 6-TG, thereby reducing the potential side effects associated with higher doses [31]. In another study, the combined use of doxorubicin, an anticarcinogenic drug, gold nanostructures, and PTT was investigated. The findings showed that this synergistic approach had superior therapeutic effects compared to individual treatments. It efficiently induced apoptosis in breast cancer cells, resulting in significantly higher cell-killing efficiency than chemotherapy or PTT alone [32].

## 3. Materials and Methods

### 3.1. Reagents Used

Chloroauric acid trihydrate (HAuCl_4_·3H_2_O) >99.9% and 2-thiouracil (C_4_H_4_N_2_OS) 97% were purchased from Sigma-Aldrich and were used without further modifications. Sodium citrate dihydrate (C_6_H_5_Na_3_O_7_·2H_2_O) >95% was obtained from Fisher Scientific. Ultrapure water (>17 MΩ) was utilized for all syntheses and solution preparations. All glassware and the magnetic stir bars were cleaned with aqua regia solution (HCl/HNO_3_ = 3:1) and rinsed with ultrapure water before use. For cell culture studies, the MDA-MB-231 cancer cell line was obtained from the American Type Culture Collection (ATCC HTB-26).

### 3.2. Synthesis of Gold Nanoparticles

Synthesis of AuNPs was performed following a published procedure from Wang et al. To prepare citrate-stabilized AuNPs, 143 mL of 0.28 mM of HAuCl_4_ aqueous solution was brought to boiling. About 7 mL of 38.8 mM of sodium citrate dihydrate were then added together, and the mixture was stirred for 15 min at 350 rpm. During this time, the color changed from purple to red, indicating that the reaction was completed. The suspension was cooled down to room temperature with additional stirring for 1 h. The resulting colloid of core AuNPs was used in the subsequent functionalization step with 2-TU.

### 3.3. Functionalization of Gold Nanoparticles (AuNPs) with 2-Thiouracil (2-TU)

The functionalization of AuNPs was conducted using a modified procedure from the literature [33]. This functionalization was achieved by adding 5 mL of a 0.8 mM of 2-TU aqueous solution to 50 mL of the aqueous colloid of core AuNPs while stirring at 350 rpm for 30 min. The resulting solution was purified by two-step centrifugation for 20 min at 13,000 rpm (15,700× *g*) for both steps. During the initial centrifugation step, any unbound 2-TU molecules in the supernatant were eliminated by micropipetting, while the pellet was washed with ultrapure water. In the subsequent step, the supernatant was removed once more, and the resulting pellet was resuspended in an equivalent volume of ultrapure water to maintain the AuNP concentration before being stored at room temperature.

### 3.4. Concentration and Purification of 2-TU-AuNPs by Comparative Centrifugation and Tangential Flow Filtration (TFF)

**Figure 8:**
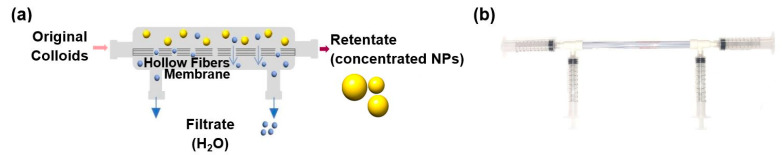
Schematic showing the working principle of tangential flow filtration (TFF): (**a**) AuNPs smaller than the pore size of the filter pass through it and are collected together with the water solvent at the filtrate side. AuNPs larger than the pore size are recirculated through the TFF system until the desired volume reduction is achieved before collection at the retentate side. (**b**) The 10 kD hollow fiber filter.

In the first approach, the resulting AuNPs were concentrated by centrifugation at 13,000 rpm (15,700× *g*) using an accuSpin 17 microcentrifuge (Fisher Scientific, Pittsburgh, PA, USA). In the second approach, colloidal 2-TU-AuNPs were concentrated by a tangential flow filtration (Figure 8a) using a 10 kD hollow fiber filter from Repligen Company (Repligen Corp., Rancho Dominguez, CA, USA) (Figure 8b).

### 3.5. Characterization of Unfunctionalized (AuNPs) and Functionalized Gold Nanoparticles (2-TU-AuNPs)

The drug loading efficiency and the concentration of 2-TU-AuNPs were determined by UV–Vis absorption spectrophotometry. These measurements were carried out in a Cary 50 dual-beam UV–Vis spectrophotometer in the 200–800 nm spectral range, using quartz cuvettes of 1 cm path length and at a spectral resolution of 1 nm. The functionalization of 2-TU-AuNPs was confirmed using Raman spectroscopy. The Raman measurements were performed using a LabRamHR 800 system (Horiba Jobin Yvon Inc., Edison, NJ, USA) equipped with 1800 and 600 grooves/mm holographic gratings. A He-Ne laser with a wavelength of 633 nm was used as the excitation line. The laser beam was focused on the colloidal samples, which were contained in cuvettes, using a high-resolution confocal Raman microscope. The laser beam spot had a diameter of 1 µm, and the spectral resolution of the system was set at 1 cm^−1^. A 100 kV Phillips 208S Transmission Electron Microscope (TEM) was employed to characterize the core size and morphology of AuNPs and 2-TU-AuNPs. Briefly, the colloidal samples (5 μL) were deposited on 300-mesh copper grids coated with a carbon support film and were allowed to air dry before imaging at 70 kV. The average core size and size distribution of nanomaterials were determined by processing the TEM images with the ImageJ 1.46R software. The hydrodynamic size and surface charge of NPs were assessed using a Zetasizer Nano ZS90 system. Disposable polystyrene cuvettes with a sample volume of 0.75–1 mL were employed for these measurements. The measuring parameters used were temperature of 25 °C; equilibration time of a sample of 120 s; dispersant (water) refractive index of 1.330 and a viscosity of 0.8872 cP; a dispersant dielectric constant of 78.5; 12 measurement runs; and a wavelength of the laser source of 532 nm. The Zetasizer system utilizes Dynamic Light Scattering (DLS) to analyze the Brownian motion of the NPs, which is then interpreted using established theories to determine their size. Additionally, the instrument measures the electrophoretic mobility of the NPs and converts it to zeta potential using software provided by Malvern Instrument (Zetasizer Software 6.12) based on Henry’s Equation (2).

(2) $$U_{E}\;=\frac{\left(2\varepsilon zf\left(Ka\right)\right)}{3\eta }$$

where U_E_ is electrophoretic mobility, ε is the dielectric constant, z is zeta potential, $f\left(Ka\right)$ is Henry’s function with a value of 1.5 for aqueous samples, and η is the viscosity of the medium.

### 3.6. Cell Culture

MDA-MB-231 breast cancer cells were cultured to confluency in Dulbecco’s modified Eagle’s Medium (DMEM), supplemented with 10% fetal bovine serum (FBS), 1% penicillin/streptomycin (10,000 U/mL penicillin and 10 mg/mL streptomycin), and 1% non-essential amino acid solution. Cells were maintained in a humidified incubator (37 °C and 5% CO_2_). The cells were subcultured once a week in a 75 cm2 flask when they reached 70–80% confluency. To subculture, the spent culture medium was removed, and the cells were washed with 10 mL of Phosphate-Buffered Saline (PBS). After that, 2 mL of trypsin:EDTA (0.25%:0.53 mM) was added, and the flask was incubated for 15 min to detach the cells. The trypsin was neutralized by adding 4 mL of the culture medium, and the cells were then centrifuged at 1200 rpm for 7 min. The supernatant was discarded, and the cells were resuspended in a fresh medium.

### 3.7. Anti-Proliferation Studies

The antiproliferative activity of AuNPs, 2-TU, and 2-TU-AuNPs was determined by the MTT (3-[4,5-dimethylthiazol-2-yl]-2,5-diphenyl tetrazolium bromide) colorimetric assay. Cell numbers were determined via trypan blue (1:1 dilution) using an automated cell counter, and only viable cells were counted to calculate concentration. Viable cells with active metabolism convert MTT into a purple-colored formazan product by enzymatic action in the mitochondria. An MDA-MB-231 breast cancer cell line was seeded into 96-well plates, at 3 × 10^4^ cells per well, and incubated in DMEM for 24 h to ensure cell adhesion to the plate. The first and second columns of 96-well plates were used as blank and control cells, respectively. From the third column, different concentrations of samples diluted in water were added to the 96-well plates (0.5–36 nM AuNPs; 0.06–0.80 mM 2-TU; 0.2–20 nM 2-TU-AuNPs). After 72 h of incubation, the MTT solution was added at concentrations of 1 mg/mL per well and was incubated for two hours. Afterward, a solution of 2-propanol with triton at 10% was added. The absorbance was measured at 570 nm and 620 nm using a microplate reader. Even though MTT and AuNPs absorb near the same wavelength, there is no interference. A study has shown that AuNPs do not absorb at the wavelength where MTT formazan is measured [34]. The half-maximal inhibitory concentration (IC_50_) was calculated using the cell viability results at different concentrations of samples. The cell viability percentage was determined by comparing it to control cells, which exhibited 100% cell viability. The cell viability of samples was calculated using the following Equation (3):

(3) $$\%\;\mathrm{cell}\;\mathrm{viability}=\frac{absorbance\;of\;treated\;cells-absorbance\;of\;blank}{absorbance\;of\;control\;cells-absorbance\;of\;blank}\;\times \;100$$

It is well known that the aggregation of AuNPs occurs in cell culture media (CCM). This aggregation in physiological media is affected by two factors: the concentration of AuNPs and the presence of serum in the CCM. A study found that monodisperse citrate-AuNPs tend to aggregate in DMEM, a commonly used cell culture medium. However, the same AuNPs remained stable in DMEM supplemented with 10% fetal bovine serum (FBS). When the AuNPs interact with proteins in the FBS, they form a protein corona that surrounds the particles, helping to stabilize them in the physiological media. The stability of AuNPs in DMEM supplemented with FBS is concentration-dependent, with aggregation occurring when the concentration exceeds 2.5 × 10^13^ NPs/mL or 41.5 nM [35].

### 3.8. Photothermal Therapy

Anti-proliferation studies were conducted by adding samples to 96-well plates. After drug dosing, the cell plates were irradiated with visible green LED light (520 nm, 1 J/cm^2^ per minute, 4000 mcd at 6.4 mW) for 10 min, followed by a 72 h incubation period to compare the antiproliferative activity between irradiated and non-irradiated nanoparticles.

### 3.9. Statistical Analysis

The experiments were repeated at least three times for each sample, and the statistical differences were determined by analysis of variance (ANOVA). The differences were regarded as significant at *p* < 0.05.

## 4. Conclusions

Citrate-stabilized AuNPs were successfully functionalized with a biologically active compound of potential anticancer activity, 2-thiouracil (2-TU). The unfunctionalized AuNPs and the functionalized 2-TU-AuNPs were then purified and characterized by UV–Vis absorption spectrophotometry, Zeta potential, and TEM. AuNPs and 2-TU-AuNPs were found to consist of monodispersed, spherical particles of a mean core diameter of 20 ± 2 nm and 24 ± 4 nm, respectively. The antiproliferative activities of AuNPs, 2-TU and 2-TU-AuNPs were examined by an MTT assay in the hormone-independent breast cancer cell line MDA-MB-231. It was established that AuNPs significantly enhanced the antiproliferative activity of 2-TU. Furthermore, the irradiation of the samples with Vis light at 520 nm decreased the half-maximal inhibitory concentration by 50%. Thus, the 2-TU drug concentration and its side effect during treatments could be significantly reduced by synergistically exploiting the antiproliferative activity of 2-TU loaded onto AuNPs and the PTT effect of AuNPs. Finally, this is a proof of concept using a low power visible radiation, demonstrating the use of 2-TU conjugated AuNPs in photothermal therapy with irradiation in the visible green LED light (520 nm), where the absorption by the tissue is minimized as compared to photothermal therapy in the UV region. To this end, 2-TU-AuNP with a plasmon in the visible region (520 nm) represents an alternative to treat triple-negative breast cancer, which has a poor prognosis; drugs to treat this type of cancer are limited and not very efficient, as well as minimize the tissue damage resulting from the UV radiation.

## 5. Future Research Directions

It is evident that the use of spherical gold nanoparticles functionalized with biologically active molecules has immense possibilities for applications in the medical field. Given that 2-TU has applications for treating hyperthyroidism and skin cancer, the subject 2-TU-AuNPs can be used as a drug to treat these diseases with a substantial reduction of side effects. Although there are other NPs geometries with potential applications in cancer, spherical AuNPs with small diameters are perfect for cancer treatment due to their easy transport inside the cells due to the increased vascularity present in cancer cells.

## Data Availability

Not applicable.

## Supplementary Materials

The following supporting information can be downloaded at: https://www.mdpi.com/article/10.3390/molecules28114453/s1, Figures S1 and S2: A graph of absorbance vs. concentration of 2-TU, and a Raman spectrum for 2-TU and 2-TU-AuNPs in the Fingerprint Region.
