# Supplementary material for: Gold Nanoparticles Functionalized with 2-Thiouracil for Antiproliferative and Photothermal Therapies in Breast Cancer Cells
Source: Molecules. 2023 May 31;28(11):4453. doi: 10.3390/molecules28114453 (PMC10254252; doi:10.3390/molecules28114453)
Supplement: Supplementary file 1 [file molecules-28-04453-s001.zip › molecules-2386556-supplementary.pdf]

Supplementary Information

Article

Gold Nanoparticles Functionalized with 2-Thiouracil for Antiproliferative and Photothermal Therapies in Breast Cancer Cells

Génesis Lorenzana-Vázquez [1], Ioana Pavel [2] and Enrique Meléndez [1,*]

[1] Department of Chemistry, University of Puerto Rico, Mayaguez Campus, Mayaguez, PR 00681, USA; genesis.lorenzana@upr.edu

[2] Department of Physical and Environmental Sciences, Texas A&M University – Corpus Christi, Corpus Christi, TX 78412, USA; ioana.pavel@tamucc.edu

* Correspondence: enrique.melendez@upr.edu

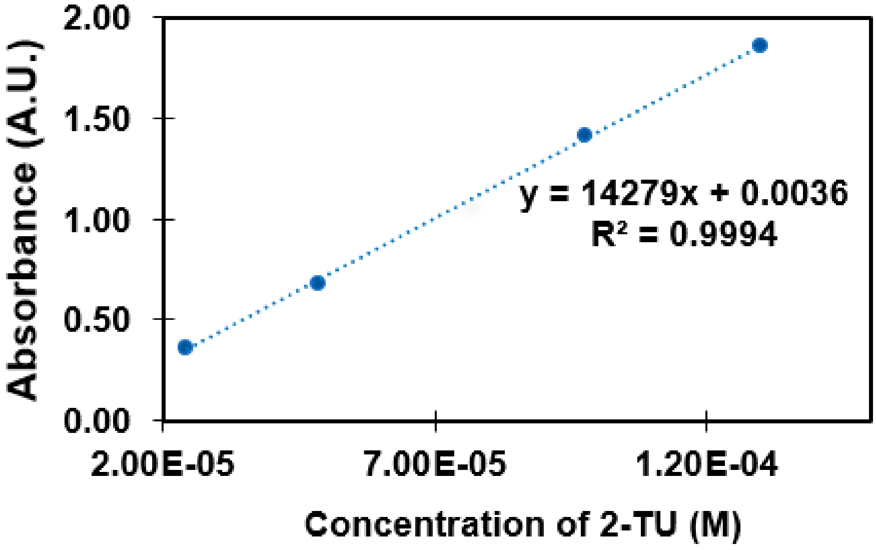

Figure S1. Graph of absorbance vs concentration of 2-thiouracil at $\lambda = 272$ nm.

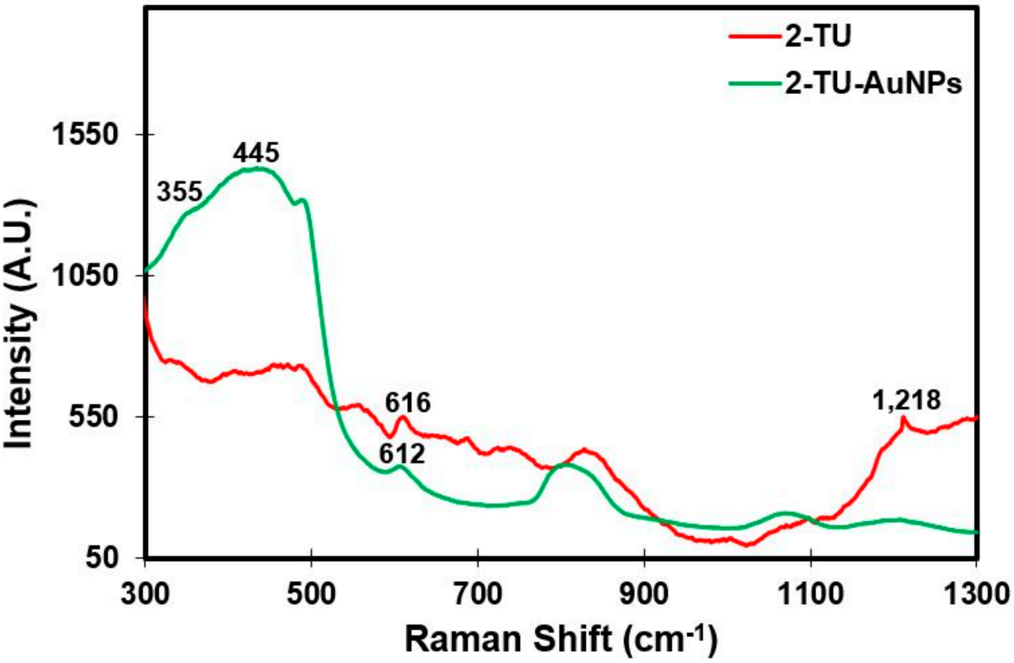

Figure S2. Raman spectra for 2-TU and 2-TU-AuNPs in the Fingerprint Region.
